# A Direct Auditory Subcortical Route to the Amygdala Associated with Fear in Humans

**DOI:** 10.1523/JNEUROSCI.1431-25.2026

**Published:** 2026-03-16

**Authors:** Emmanouela Kosteletou-Kassotaki, Martina T. Cinca-Tomás, Federico Varriano, Guadalupe Soria, Alberto Prats-Galino, Judith Domínguez-Borràs

**Affiliations:** ^1^ Department of Clinical Psychology and Psychobiology, University of Barcelona, Barcelona 08035, Spain; ^2^Institute of Neurosciences, University of Barcelona, Barcelona 08035, Spain; ^3^Laboratory of Surgical Neuroanatomy, Faculty of Medicine, University of Barcelona, Barcelona 08036, Spain; ^4^Institut d'Investigacions Biomèdiques August Pi i Sunyer (IDIBAPS), Barcelona 08036, Spain

**Keywords:** amygdala, auditory, diffusion-weighted imaging, emotion, fear, medial geniculate body

## Abstract

Rapid and efficient fear processing is essential for survival. In vision, this function is supported by a well-characterized subcortical pathway consisting of direct projections from the pulvinar of the thalamus to the amygdala in the human brain. In contrast, the existence of an analogous shortcut for fear in audition has been demonstrated in nonhuman animals but remains unconfirmed in humans. To address this question, we used probabilistic streamline tractography and fixel-based analysis on diffusion-weighted images from Human Connectome Project participants of either sex to reconstruct candidate auditory subcortical pathways and examine their associations with affective and auditory behavioral measures. Our findings revealed a robust white matter tract connecting the inferior colliculus to basolateral amygdala via the medial geniculate body (MGB) of the thalamus. Remarkably, higher fiber density in this tract was associated with better hearing ability in noise and increased self-reported fearfulness, supporting its role in auditory and affective function. Conversely, a control analysis of the core thalamocortical pathway from ventral MGB to primary auditory cortex, representing the main route for auditory processing, was associated with auditory ability but not with affective measures. These findings provide previously unreported evidence for an auditory colliculo-geniculo-amygdala "low road" in humans, aligning with evolutionarily conserved pathways for fear described in nonhuman species.

## Significance Statement

Rapid fear processing is crucial for survival. While a visual subcortical “low road” for fear is well characterized in humans, the existence of an analogous human auditory shortcut remains undetermined. Using diffusion magnetic resonance imaging tractography, we provide evidence for a white matter tract connecting the inferior colliculus to basolateral amygdala via the medial geniculate body of the thalamus, which is associated with hearing ability and self-reported fearfulness. Our findings provide novel evidence for an auditory colliculo-geniculo-amygdala direct route in humans, revealing an evolutionarily conserved pathway for fear previously described in nonhuman species.

## Introduction

Vertebrates, including humans and other primates, rely on rapid fear and threat processing as a critical evolutionary adaptation for survival. Neural systems facilitating sensory and fear processing play a central role in this process. In humans, one of the most extensively investigated pathways serving this ability is the so-called visual “low road” to the amygdala (LeDoux, 1996; McFadyen et al., 2020), a phylogenetically ancient route (Carr, 2015) that presumably involves direct projections from the retina to the superior colliculus, from there to the pulvinar of the thalamus, and from pulvinar to basolateral amygdala (BLA), bypassing the primary visual cortex. This pathway enables rapid and crude transmission of visual information to the amygdala and mediates several fear-related processes, such as unconscious fear processing in blind patients with bilateral occipital damage (Tamietto and De Gelder, 2010) and abundant fear-associated behaviors (Vuilleumier, 2005; Celeghin et al., 2019; McFadyen et al., 2020; Domínguez-Borràs and Vuilleumier, 2022), with abnormal function reported in higher anxiety individuals (Nakataki et al., 2016; McFadyen et al., 2020). Evidence for this visual pathway has been reported across multiple species, including rodents, nonhuman primates, and humans (Doron and LeDoux, 1999; Day-Brown et al., 2010; Tamietto et al., 2012; Rafal et al., 2015; Wei et al., 2015; Elorette et al., 2018; McFadyen et al., 2019; Kragel et al., 2021).

Critically, the existence of a similar subcortical fear pathway in humans along the auditory system remains unknown, despite extensive evidence in nonhuman animals. Studies in rodents and other mammals have consistently described direct projections to BLA via the auditory medial geniculate body (MGB) of the thalamus (Aggleton et al., 1980; LeDoux et al., 1990; Bordi and LeDoux, 1994) which in turn receives major auditory inputs from the inferior colliculus (IC; Bordi and LeDoux, 1994). This auditory route is known to have a key role in emotional and motivational processes, mediating fear in various species (LeDoux et al., 1984; Shinonaga et al., 1994; Khalil et al., 2023).

Here, we tested whether a subcortical auditory pathway associated with fear is also present in humans, by applying diffusion-weighted imaging (DWI) tractography and fixel-based analysis (FBA) to data from the Human Connectome Project (HCP; *N* = 200; Raffelt et al., 2012; Raffelt et al., 2017; Dhollander et al., 2021). Specifically, we tested anatomical evidence for a candidate pathway connecting the IC to the BLA via MGB. This corresponds to the subcortical pathway originally described in animal models (LeDoux et al., 1984; Doron and LeDoux, 1999), where auditory information is relayed from IC to MGB and subsequently to BLA. To evaluate its functional significance, we further investigated its linear associations with auditory and fear-related behavioral measures available in the HCP dataset. As an auditory pathway for fear, we hypothesized that the properties of this tract would predict both auditory ability and fear-related function.

For completeness, we also examined two subcortical pathways putatively associated with auditory processing and fear. First, we tested a connection between IC and BLA (bypassing MGB), previously described in isolated reports in tinnitus patients (Crippa et al., 2010). Second, we evaluated an amygdala tract that encompassed pulvinar segments previously reported to receive prominent auditory and multisensory input. Specifically, while its inferior and lateral segments have consistently been characterized as predominantly visual (Grieve et al., 2000; Bourgeois et al., 2020), its anterior and medial segments may be particularly associated with auditory or multisensory function in nonhuman primates (Homman-Ludiye and Bourne, 2019; Vittek et al., 2023; Froesel et al., 2024). An auditory pulvino-amygdala tract for fear would be consistent with recent findings suggesting that amygdala projections from the pulvinar (with no distinction of its subregions in this case) might also mediate auditory emotion (Kragel et al., 2021).

Finally, as a control tract, we examined the thalamocortical auditory pathway connecting the ventral MGB (vMGB) to primary auditory cortex (PAC), hypothesizing that it would be associated with auditory ability but not affective function (Lee, 2012).

## Materials and Methods

### Participants

All data were acquired from the publicly available HCP (https://www.humanconnectome.org/) S1200 release, containing data from 1,206 healthy young adult participants, collected during the years 2012–2015 (Van Essen et al., 2013). Out of these participants, 200 were selected for our study (mean age = 28.78 ± 3.87 years, 107 females, 93 males) with complete magnetic resonance imaging (MRI) and diffusion MRI (dMRI) data, right-handed, negative alcohol or drug consumption tests, normal hearing ability, no history of major depressive episodes or neurological conditions, and within normal anxiety levels (mean = 52.69 ± 4.02), assessed by the ASR DSM Anxiety Problems gender and age weighted test, to ensure a homogeneous sample and avoid outliers. Access permission to the data was obtained from the Washington University–University of Minnesota Consortium of the Human Connectome Project.

### dMRI processing

#### dMRI acquisition

The HCP data were acquired on a customized Siemens Skyra 3 T scanner with a 32-channel head coil. Two separate averages of the structural T1-weighted 3D MPRAGE images were acquired (voxel size, 0.7 mm isotropic; FOV, 224 mm; matrix, 320; 256 sagittal slices; TR, 2,400 ms; TE, 2.14ms; TI, 1,000 ms; FA, 8°; bandwidth, 210 Hz per pixel; echo spacing, 7.6 ms). Diffusion-weighted images were acquired in the same scanner using a multiband multishell spin-echo EPI sequence (voxel size, 1.25mm isotropic; TR, 5,520 ms; TE, 89.5 ms; FA, 78°; FOV, 210 mm; three shells with *b* values of 1,000, 2,000, and 3,000 s/mm^2^), with 90 directions per shell and 6 interspersed baseline images (*b* = 5 s/mm^2^) for each shell (Glasser et al., 2013).

#### dMRI preprocessing

We used the minimally preprocessed structural and diffusion data provided by the HCP. For the structural images, this included gradient nonlinearity distortion correction, bias field correction, segmentation, and smoothing (Glasser et al., 2013). For the diffusion images, intensity normalization of the mean *b*0 image, field distortion correction, and eddy current inhomogeneities correction were applied (Andersson et al., 2003; Andersson and Sotiropoulos, 2016). All further dMRI processing and tractography analysis was performed on MRtrix 3.0.4. (Tournier et al., 2019). Response functions for gray matter, white matter, and cerebrospinal fluid (CSF) were estimated using the multishell multitissue constrained spherical deconvolution (MSMT-CSD) algorithm for each participant and averaged to estimate the response functions per tissue type (Jeurissen et al., 2014). Using these average response functions, we then produced individual multitissue fiber orientation distributions (FODs) with the MSMT-CSD algorithm. Finally, we performed joint bias field correction and global intensity normalization of the multitissue compartment parameters using the mtnormalise command in MRtrix3.

### Regions of interest

Based on our hypotheses, to reconstruct our candidate auditory subcortical pathways to the amygdala based on animal literature (LeDoux et al., 1984; Doron and LeDoux, 1999), selected key regions of interest (ROIs) included the IC, the MGB of the thalamus, the anterior and medial pulvinar (PUL), and the BLA. The inclusion of the IC enabled us to reconstruct a more complete subcortical route, as originally described in animal models (LeDoux et al., 1984; Doron and LeDoux, 1999). It also allowed us to compare a direct IC–BLA with an IC–MGB–BLA pathway. The anterior and medial pulvinar were chosen as the most plausibly involved in auditory but also multisensory processing, compared with the predominantly visual inferior pulvinar (Homman-Ludiye and Bourne, 2019; Vittek et al., 2023; Froesel et al., 2024). In turn, BLA is the main amygdala subregion associated with the processing of sensory input in fear processes (LeDoux, 1996). For the control analysis, we used two additional ROI masks, which included the vMGB and the PAC. Both ROIs belong to the auditory thalamocortical pathway, which represents the primary route for auditory processing in the mammalian brain. We therefore hypothesized that this pathway would be associated with auditory ability, but not with affective processes, as it does not involve projections to the amygdala.

For our ROIs, we selected different atlases offering optimal anatomical precision for each structure. For the smallest ROIs, namely, IC and vMGB, we used ultrahigh-resolution 7 T MRI segmentations (Mihai et al., 2019; Bianciardi et al., 2023), which provide high in vivo anatomical precision in small-volume regions. The MGB, BLA, and PAC ROIs were obtained from standard cytoarchitectonic probability maps providing high anatomical fidelity. The MGB mask was defined using cytoarchitectonic maps of the human metathalamus, as published by Kiwitz et al. (2022) and available at https://www.ebrains.eu/. Please note that thalamo-amygdala projections have been reported to originate predominantly in the medial portion of MGB in nonhuman species (LeDoux et al., 1984). However, given its small size and the lack of validated masks encompassing this subregion, we used a whole MGB mask for these analyses, which provides the most reliable anatomical definition for dMRI analyses. Anatomy Toolbox was employed to acquire probabilistic cytoarchitectonically verified maps of both the BLA and the PAC (Amunts et al., 2005; Eickhoff et al., 2005). Finally, PUL subdivisions were derived from a parcellated pulvinar atlas (Barron et al., 2015), as used in McFadyen et al. (2019), which delineates five distinct pulvinar clusters based on coactivation profiles observed in a large functional MRI dataset and, notably, shows correspondence with cytoarchitectonic parcellations. We combined the anterior and medial PUL segments of this parcellated mask using the FSL software (Jenkinson et al., 2012). All ROIs were defined in MNI space and registered to each participant's native diffusion space using FSL (Jenkinson et al., 2002, 2012; Greve and Fischl, 2009) prior to tractography.

### dMRI analysis

In this study, we used two different approaches for analyzing dMRI data and exploring the architecture of the white matter tracts of interest. First, we performed probabilistic bidirectional streamline tractography between our ROIs, which uses a Bayesian method to account for one or more FODs in each voxel (Tournier et al., 2012, 2019). This approach allowed us to reconstruct and quantify the macroscale structural connection between ROIs, by estimating the density of reconstructed streamlines along the pathways. Values corresponding to the streamline fiber density were acquired for each pathway after the application of Spherical-Deconvolution Informed Filtering of Tractograms version 2 (SIFT2) method, which weights each streamline by a cross-sectional area multiplier directly related to the underlying data (Smith et al., 2015). As a complementary estimation of fiber density, we applied FBA to our data, which allowed us to assess microstructural differences in fiber density at the fixel level, an individual fiber population within a voxel, providing a more localized and biologically specific estimation of white matter integrity. FBA is based on the observation that at high *b* value, the diffusion signal is overwhelmingly driven by the intra-axonal compartment, and since the FOD function (fODF) we extract with CSD is directly proportional to the dMRI signal, the amplitude of our fODF peaks provides an estimate of the apparent density of axonal fibers (Raffelt et al., 2015, 2017). This dual approach allowed us to both map our anatomical tracts of interest at the macrostructural level and also evaluate their microstructural variability, which may in turn reflect individual differences in the capacity of axonal pathways (Raffelt et al., 2012).

#### Streamline tractography

The MRtrix software was used to carry out bidirectional streamline probabilistic tractography. We implemented the iFOD2 algorithm to initially produce a whole-brain tractogram with 10 million streamlines generated and thresholded using a FOD amplitude cutoff of 0.06. The anatomically constrained tractography approach, through which a standard five-tissue segmentation (cortical gray matter, subcortical gray matter, white matter, CSF, pathological tissue) of the T1 image is created, as well as the SIFT2 method, was applied to minimize false-positive or false termination streamline generation (Smith et al., 2012, 2015). In the same line, we produced ROI-to-ROI tractograms planting a seed point 25,000 times in each voxel of the seeding ROI, which were finally combined with the whole-brain tractogram for the quantification of the connectivity between the ROIs of interest. Using these methods, we initially reconstructed our candidate subcortical pathways (IC–MGB–BLA, IC–BLA, anterior/medial PUL–BLA) and calculated the fiber density of each connection. Additionally, as a control test, we applied the same analysis to the thalamocortical pathway (vMGB–PAC) to evaluate whether this primary auditory route, which does not involve direct amygdala projections, correlated with fear-associated measures.

#### Fixel-based analysis

To achieve a more detailed and biologically specific understanding of the microstructural connections within the white matter, we implemented FBA, with the methodology outlined in the MRtrix3 documentation for multitissue CSD data. After the FODs were computed and intensity normalized for each participant, we generated a group-specific FOD template using a subset of 20 participants (10% of our total sample). The FOD image of each subject was then registered to the FOD template. Additionally, a template mask from the brain masks of each participant and a template white matter analysis fixel mask were computed. After completing these steps, we mapped each tractogram of interest (IC–MGB–BLA, IC–BLA, anterior/medial PUL–BLA, vMGB–PAC) to the fixel analysis mask and finally calculated the apparent fiber density for each subject across these specific pathways. Apparent fiber density reflects the intra-axonal volume of white matter fibers within a specific orientation in a voxel (a “fixel”), as a microstructural index of axonal density (Raffelt et al., 2017; Dhollander et al., 2021).

### Correlations with behavioral data

Our primary goal was to identify direct subcortical tracts to the amygdala that may be both auditory in nature and associated with affective processing, particularly with fear-related function. Because the dataset did not include direct measures of auditory emotion function (as it does for visual emotion), we relied on the most relevant available indicators. Thus, we hypothesized that fiber structure and density of these tracts would correlate, on the one hand, with individual hearing ability measures of the 200 participants and, on the other, with their individual scores from affective measures.

To test for auditory perceptual abilities, we selected scores from the NIH Toolbox Words-in-Noise Test (hearing ability in noise), as it provided the closest measure of auditory perceptual function available in the dataset, linked to subcortical encoding of acoustic information (Parbery-Clark et al., 2011). The choice was motivated by evidence that the auditory thalamus sends auditory sensory input to the amygdala (Armony et al., 1997) and that speech-in-noise is associated with subcortical auditory processing in MGB (von Kriegstein et al., 2008; Chandrasekaran et al., 2009; Bernstein et al., 2016), beyond thalamocortical processing (Mihai et al., 2019). In this test, participants were asked to identify words presented to each ear individually in a background of increasingly loud noise, thereby reducing the signal-to-noise ratio. A threshold score is calculated for each ear in decibels of signal-to-noise ratio (dB S/N) and combined into a single composite score. The range of possible scores for each ear is −2.0 to 26.0 dB S/N, with lower scores indicating better performance and higher scores suggesting potential hearing difficulties (Zecker et al., 2013). In turn, to examine fear-related function in our participants, we used the NIH Toolbox Fear-Affect Survey (Fearfulness), a computerized adaptive test derived from the PROMIS Anxiety Item Bank. This survey is part of a broader Negative Affect domain assessing fear, anxiety, anger, and sadness. For our analyses, we focused specifically on the fearfulness component of the score, measuring self-reported feelings of fear and anxious distress over the past 7 d (thus reflecting a fearful state). This component is normed and validated as a fear-related construct and is based on the fact that fear is best characterized by anxiety-related autonomic arousal and perceptions of threat. Scores are unadjusted *T*-scores (mean = 50; SD = 10), with ≤40 indicating low fearfulness and ≥60 indicating high fearfulness (Pilkonis et al., 2011, 2013). The scale has excellent internal consistency (Cronbach's *α* = 0.95), strong convergent validity (*r* = 0.8), and good confirmatory factor analysis indices (comparative fit index, 0.995; root mean square error of approximation, 0.086), supporting the reliability of the scores in measuring fear-specific processes (Salsman et al., 2013).

For completeness, we additionally examined scores from two visual emotion tasks available in the dataset, which are associated with facial expression recognition. Specifically, we selected accuracy and response times in milliseconds from an in-scanner emotion-matching task involving fearful faces, as well as recognition accuracy for fearful faces from the out-of-scanner Penn Emotion Recognition Task. Median response times from the Penn Emotion Recognition Task were excluded, as they reflect responses across all emotions, not only fearful faces. In the former, participants matched faces presented at the top and the bottom of the screen, with the faces displaying either anger or fear. In the latter, the participants identified the emotional expression of fearful faces. These visually based tasks were applied to all tracts of interest (IC–MGB–BLA, IC–BLA, anterior/medial PUL–BLA, vMGB–PAC) to additionally test whether any of these tracts might also be associated with visual emotion processing, as previous research reported that fiber density within the pulvinar–amygdala tract (corresponding to the visual subcortical pathway), correlated with fearful-face recognition from similar tasks (McFadyen et al., 2019). Response time in visual emotion tasks is a relevant indicator of the emotional response, correlating with amygdala activity (Domínguez-Borràs et al., 2019). Thus, we hypothesized that, if fiber density within our reconstructed tracts correlated with scores in these visual tests, they may additionally have a role in visual emotion. Descriptive statistics of these behavioral measures are reported in Table S1.

### Statistical analyses

We explored the relationship between these behavioral measures and the white matter metrics derived from both the streamline tractography (streamline fiber density) and the FBA frameworks (apparent fiber density) using linear mixed-effect models. Separate models were run for each tract (IC–MGB–BLA, IC–BLA, anterior/medial PUL–BLA, vMGB–PAC) and metric. Initially, all continuous predictors were standardized using *z*-score transformation to facilitate model convergence, improve interpretability, and allow comparison of effect sizes across variables. Fixed effects (predictors) included hemisphere (within-subject factor, left vs right), hearing ability in noise, fearfulness, accuracy scores and response times from the in-scanner emotion-matching task, and out-of-scanner fearful-face recognition accuracy scores. Including behavioral scores as predictors of the fiber allowed us to test whether individual differences in these variables are reflected in the underlying micro- and macrostructural properties of the subcortical tracts. This approach is consistent with previous work (McFadyen et al., 2019) and reduces the risk of inflating multicollinearity when modeling multiple behavioral outcomes from a single neural metric. Age and handedness were included as controlling covariates. To model the dependency between hemisphere measurements within individuals, we included participants as a random intercept. For predictors with significant main effects, we tested hemisphere-specific modulation by adding individual interaction terms (e.g., predictor × hemisphere) and comparing models via likelihood ratio tests. A model including all possible interactions showed no significant improvement in fit, so we retained only interactions with significant main effects. Simple slopes (estimated marginal trends) for retained interactions were computed using the emmeans package (Lenth, 2025) to quantify interhemispheric differences. Analyses used restricted maximum likelihood with *α* = 0.05, and Bonferroni’s correction was applied when multiple interactions were tested within a tract/metric. All models were implemented in R (v4.3.0) with the lme4 package (Bates et al., 2015). Finally, to evaluate whether the produced streamline counts were reconstructed significantly above chance, we conducted paired two-sided *t* tests to compare the streamlines of each pathway with those produced by a null distribution algorithm (Morris et al., 2008).

## Results

**Figure 1.**
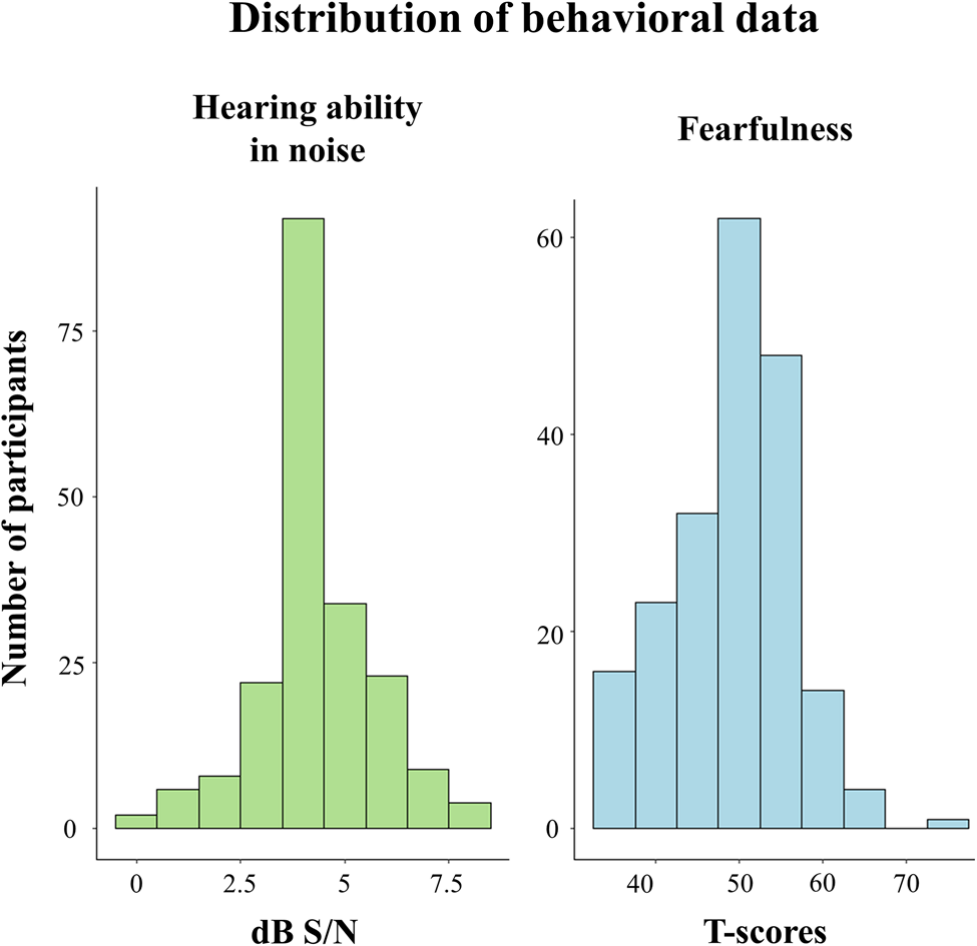
Distribution of raw behavioral scores. Left, Hearing ability in noise. The *y*-axis represents the number of participants (frequency), and the *x*-axis shows the hearing ability in noise in dB S/N. Scores range from −2.0 to 26.0 dB S/N, with lower scores indicating better performance. Right, Fearfulness. The *y*-axis represents the number of participants (frequency), and the *x*-axis shows the unadjusted *T*-scores (mean = 50; SD = 10). Scores ≤40 indicate low levels of fearfulness, and scores ≥60 indicate high levels of fearfulness.

Our goal was to test the anatomical evidence for an auditory subcortical pathway to the amygdala in humans, similar to the visual “low road” for fear, based on previous findings in nonhuman animals. For that, we reconstructed distinct candidate pathways, calculated their streamline fiber density and FBA apparent fiber density, and tested their relationships with hearing ability and affective-related behavioral predictors. Figure 1 displays the distributional properties of hearing ability in noise and fearfulness, while the distributions of the remaining behavioral measures are presented in Figure S1.

### A reconstructed IC–MGB–BLA pathway in humans, with fiber density in the tract associated with hearing ability and fearfulness

**Figure 2.**
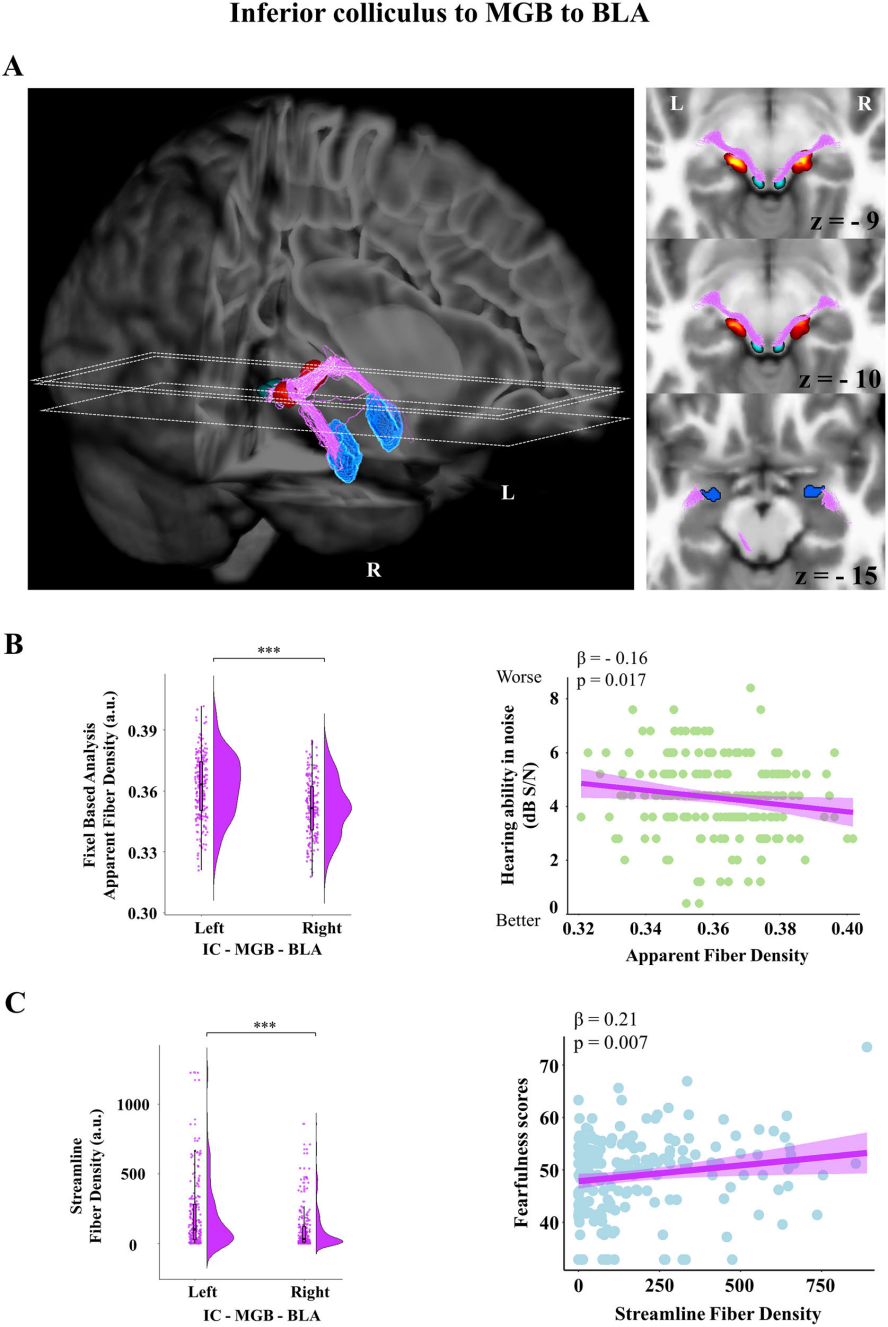
Probabilistic tractography reconstruction of the auditory subcortical route to the amygdala, via an IC to MGB pathway. ***A***, Left, 3D probabilistic tractography reconstruction and (***A***, right) axial slices at stereotaxic levels *z* = −9, −10, and −15 in a representative subject. Dashed lines indicate the correspondence between each slice and its position in the 3D rendering. Fibers reconstructed seeding from IC, passing through the MGB and terminating to the BLA (IC in light blue, MGB in red, BLA in dark blue, streamlines in purple). ***B***, Left, Distribution of the apparent fiber density from FBA showing leftward hemispheric asymmetry (*p* < 0.001). ***B***, Right, Apparent fiber density for the left IC–MGB–BLA tract correlated negatively with participants’ hearing ability in noise thresholds, expressed in dB S/N. Thus, better hearing ability was associated with higher apparent fiber density in this tract, supporting its association with auditory function. ***C***, Left, Fiber density distribution from probabilistic streamline tractography showing leftward hemispheric asymmetry (*p* < 0.001). ***C***, Right, Fiber density for the left IC–MGB–BLA tract correlated positively with fearfulness scores (≤40 = low fearfulness; ≥60 = high fearfulness), indicating that higher fiber density correlated with more feelings of fear in this tract.

We generated streamlines that seeded from IC, passed by the MGB of the thalamus and terminated in the BLA. These revealed IC–MGB–BLA connections (number of streamlines; left, *M* = 359.66; SD = 607.81; right, *M* = 169.87; SD = 325.19), with 10 and 22 participants having zero fibers for the left and right pathway, respectively (Fig. 2*A*). All connections exhibited streamline counts significantly different than those expected by chance (left, *t*_(199)_ = 5.5; *p* < 0.001; right, *t*_(199)_ = 4.1; *p* < 0.001).

In turn, a main effect of the hemisphere was found in the apparent fiber density obtained from our FBA, informing about microstructural properties, with its values being higher in the left hemisphere compared with the right (left, *M* = 0.36; SD = 0.02; right, *M* = 0.35; SD = 0.01; *b* = −0.01; SE = 0.0005; *t*_(193)_ = −19.92; *p* < 0.001; 95% CI [−0.011, −0.009]; standardized *β* = −0.65). A significant relationship between hearing ability in noise and apparent fiber density was found, such that better hearing ability in noise was associated with higher apparent fiber density values in the IC–MGB–BLA pathway (*b* = −0.003; SE = 0.0001; *t*_(211)_ = −2.40; *p* = 0.017; 95% CI [−0.0046, −0.0005]; standardized *β* = −0.16). While the interaction between hemisphere and hearing ability in noise was not significant (*b* = 0.0006; SE = 0.0005; *t*_(193)_ = 1.23; *p* = 0.22; 95% CI [−0.0004, 0.0017]; standardized *β* = 0.04), indicating no remarkable difference between hemispheres in the strength of this association, further exploratory analysis suggested that hearing ability in noise may be more strongly associated with apparent fiber density on the left (*β* = −0.16; 95% CI [−0.005, −0.0005]; *p* = 0.017) relative to the right hemisphere (*β* = −0.12; 95% CI [−0.004, 0.0002]; *p* = 0.074; Fig. 2*B*). Thus, better performance in this auditory test was associated with higher fiber density in this pathway, which in turn supports a role of the reconstructed tract in auditory perceptual ability (Parbery-Clark et al., 2011).

In turn, fiber density obtained from our streamline tractography confirmed again a leftward hemispheric lateralization (left, *M* = 197.38; SD = 247.32; right, *M* = 97.54; SD = 150.84; *b* = −81.77; SE = 13.38; *t*_(188)_ = −6.11; *p* < 0.001; 95% CI [−107.99, −55.55]; standardized *β* = −0.45). After removing four participants with extreme outlying standardized residuals (*z*-score threshold ± 3.5), a positive relationship between fearfulness scores and streamline fiber density in the IC–MGB–BLA tract was found (*b* = 37.64; SE = 12.86; *t*_(313)_ = 2.93; *p* = 0.004; 95% CI [12.43, 62.85]; standardized *β* = 0.21), indicating that more emotions of fear were associated with higher fiber density values. While this association did not differ by hemisphere, as evidenced by the hemisphere × fearfulness interaction (*b* = −16.55; SE = 13.4; *t*_(188)_ = −1.24; *p* = 0.22; 95% CI [−42.8, −9.71]; standardized *β* = −0.09), follow-up analyses revealed a stronger effect of fearfulness on fiber density in the left hemisphere (*β* = 0.21; 95% CI [8.67, 66.6]; *p* = 0.007), relative to the right (*β* = 0.12; 95% CI [−8.98, 47.7]; *p* = 0.2; Fig. 2*C*). Thus, the higher the fearfulness scores of the participants, the higher the streamline fiber density in the left IC–MGB–BLA tract. This supports a contribution of the macrostructural density of this tract to fear-related processes. No significant main effects were found between fiber density in the IC–MGB–BLA pathway and accuracy scores or response times from any of the visual emotion recognition tasks tested. Please note that similar behavioral results were observed in a control analysis connecting the MGB to BLA, without the inclusion of IC, despite the higher number of streamlines in the MGB–BLA tract compared with the IC–MGB–BLA, reflecting the stricter tract reconstruction criterion imposed by the additional inclusion ROI (Supplemental Material, Text S1, Fig. S2).

### No evidence for a direct IC–BLA connection in humans

**Figure 3.**
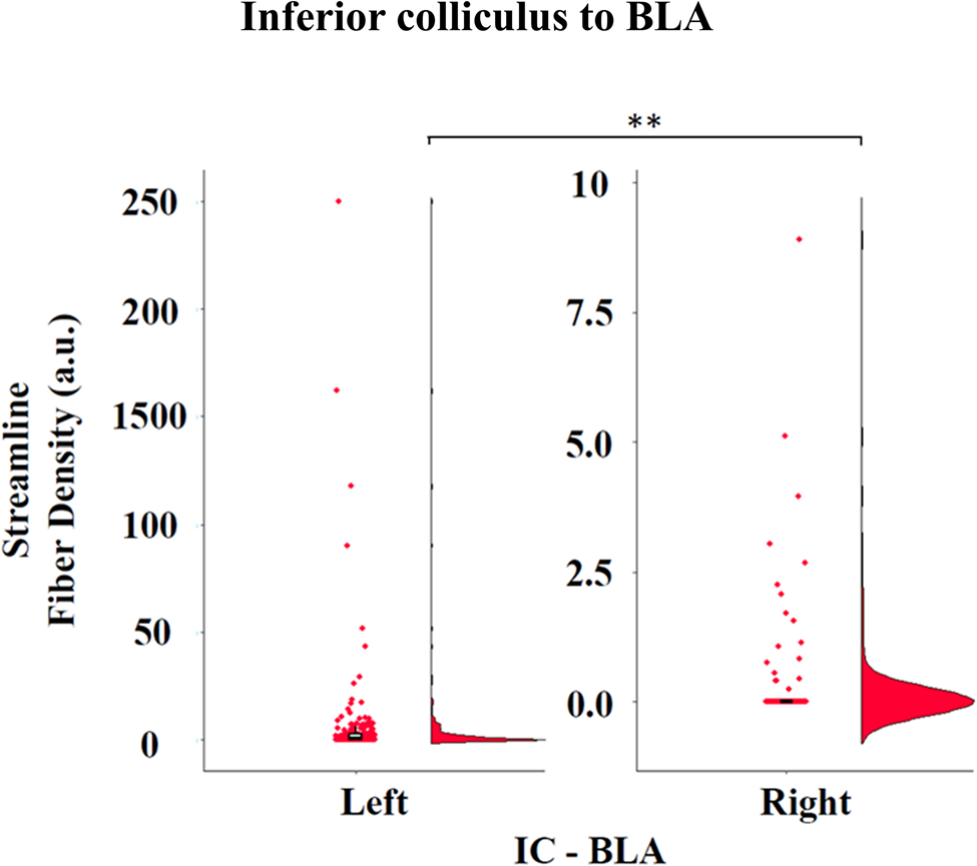
Streamline-based fiber density for the direct IC–BLA pathway. The distribution shows substantially lower fiber density values compared with the IC–MGB–BLA pathway, with a significant leftward hemispheric asymmetry (*p* = 0.002). Most participants showed zero or near-zero streamlines, supporting the absence of a meaningful direct anatomical connection between IC and BLA.

A direct connection between the IC and BLA was also reconstructed (Crippa et al., 2010), using the MGB ROI as an exclusion mask, to avoid the inclusion of streamlines passing through MGB before reaching BLA. A direct connection was rarely observed, with a large number of participants showing no detectable streamlines directly connecting these regions (number of streamlines; left, *M* = 11.68; SD = 55.64; right, *M* = 0.22; SD = 1.02). Specifically, 100 and 182 participants had zero streamlines in the left and right hemispheres, respectively. Comparison with the null distribution algorithm revealed a significantly higher number of streamlines generated by the null distribution bilaterally (left, *t*_(199)_ = −9.1; *p* < 0.001; right, *t*_(199)_ = −8.2; *p* < 0.001). The distribution of streamline fiber density for the IC–BLA pathway is shown in Figure 3. A significant hemispheric lateralization was observed, with higher values in the left compared with the right hemisphere (left, *M* = 5.68; SD = 23.8; right, *M* = 0.19; SD = 0.87; *b* = −5.27; SE = 1.68; *t*_(392)_ = −3.13; *p* = 0.002; 95% CI [−8.57, −1.97]; standardized *β* = −0.31). As expected, streamline density values for the IC–BLA pathway were substantially lower than those of the IC–MGB–BLA pathway. For a direct visual comparison of both tract distributions, see Figure S3. Finally, no significant correlations were found between fiber density metrics of the IC–BLA tract (derived from both streamline tractography and FBA) and any behavioral measures of interest. Together, these results support the absence of a direct IC–BLA pathway in the human brain and provide further evidence that the IC–MGB–BLA reconstructed pathway was not driven by artifactual streamlines across anatomically unrelated structures.

### Association of an anteromedial PUL–BLA tract with visual emotion recognition, but not hearing ability or fearfulness

**Figure 4.**
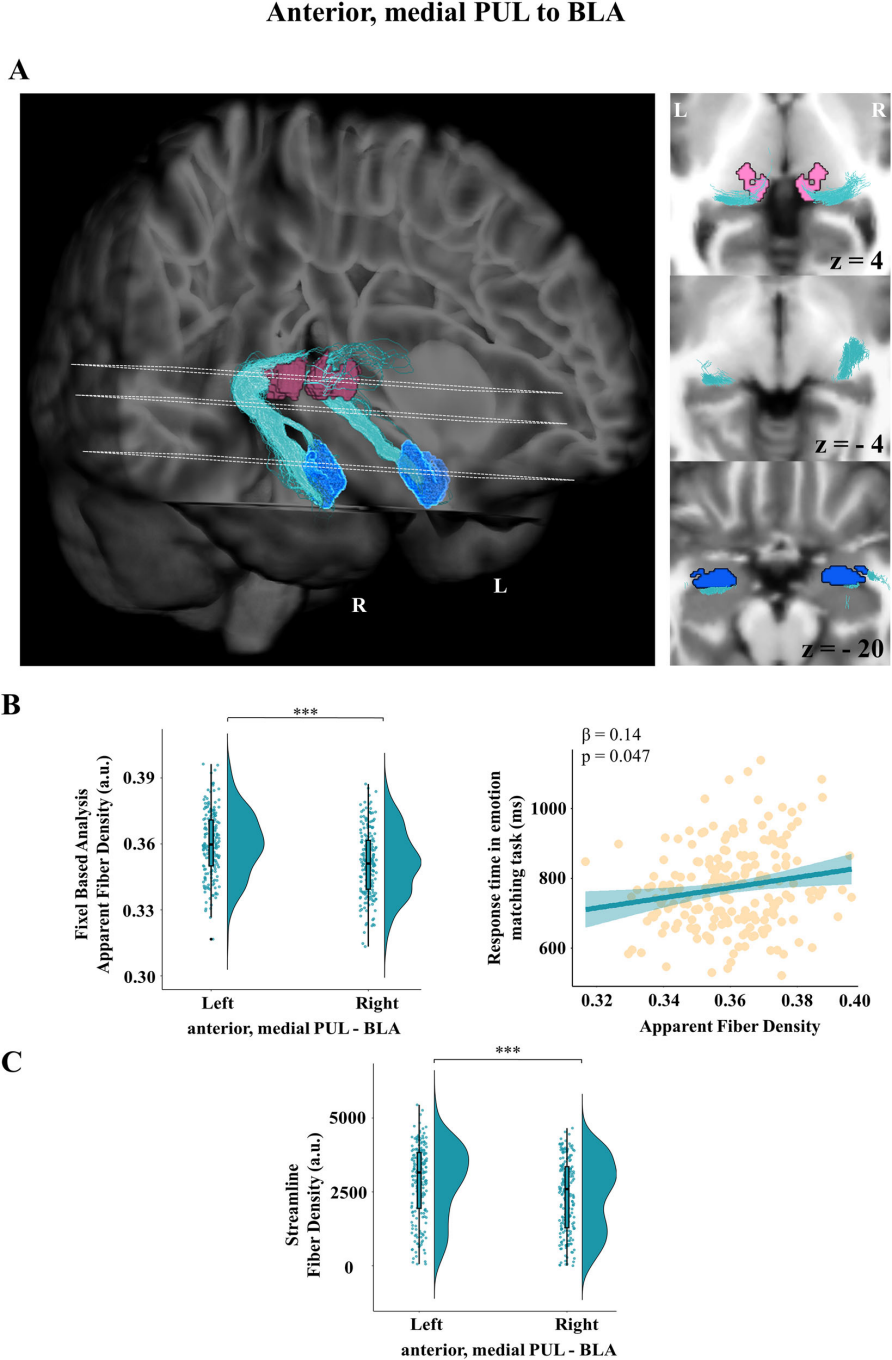
Probabilistic tractography reconstruction of the subcortical anterior, medial pulvinar connection to the BLA. ***A***, Left, 3D probabilistic tractography reconstruction and (***A***, right) axial slices at stereotaxic levels *z* = 4, −4, and −20 in a representative subject. Dashed lines indicate the correspondence between each slice and its position in the 3D rendering. Fibers reconstructed between the anterior and medial segments of the pulvinar (PUL) and the BLA (PUL in pink, BLA in dark blue, streamlines in turquoise). ***B***, Left, Apparent fiber density distribution from FBA showing a left-hemispheric lateralization (*p* < 0.001). ***B***, Right, Apparent fiber density for the left anteromedial PUL–BLA tract positively correlated with response times (milliseconds) in emotion-matching task, indicating its potential involvement in visual emotion recognition. ***C***, Fiber density distribution from probabilistic streamline tractography showing a left-hemispheric lateralization (*p* < 0.001).

The anterior and medial portions of the PUL (Froesel et al., 2021; Vittek et al., 2023) were robustly connected with BLA (number of streamlines; left, *M* = 9,811.9; SD = 4,652.12; right, *M* = 8,351.72; SD = 5,261.93), with all participants having at least one connecting streamline (Fig. 4*A*). The comparison between the reconstructed streamlines and the null distribution algorithm was statistically significant bilaterally, with both hemispheres having more reconstructed streamlines than the noise algorithm (left, *t*_(199)_ = 19.9; *p* < 0.001; right, *t*_(199)_ = 15.8; *p* < 0.001).

A significant main effect of hemisphere was observed for fixel-based apparent fiber density, with lower values in the right hemisphere compared with the left (left, *M* = 0.36; SD = 0.01; right, *M* = 0.35; SD = 0.01; *b* = −0.0087; SE = 0.0004; *t*_(193)_ = −20.45; *p* < 0.001; 95% CI [−0.009, −0.008]; standardized *β* = −0.56). Response time on the in-scanner emotion-matching task, involving fearful faces, was a significant predictor of fixel-based apparent fiber density in this tract, such that higher response times were associated with increased fiber density (*b* = 0.0022; SE = 0.0011; *t*_(202)_ = 2.0; *p* = 0.047; 95% CI [0.00008, 0.004]; standardized *β* = 0.14). Nevertheless, the interaction between response time and hemisphere was not significant (*b* = −0.0001; SE = 0.0004; *t*_(193)_ = −0.35; *p* = 0.73). A follow-up analysis indicated that the association between response times on the emotion-matching task and fixel-based apparent fiber density was driven predominantly by the left hemisphere (*β* = 0.14; 95% CI [0.00003, 0.004]; *p* = 0.047), relative to the right (*β* = 0.13; 95% CI [−0.0001, 0.004]; *p* = 0.06; Fig. 4*B*). This points to a potential involvement of the reconstructed tract in visual emotion recognition.

A left to right hemispheric lateralization was also present in the streamline fiber density of the anteromedial PUL–BLA pathway (left, *M* = 2,867.28; SD = 1,243.55; right, *M* = 2,384.15; SD = 1,231.36; *b* = −464.12; SE = 78.21; *t*_(193)_ = −5.94; *p* < 0.001; 95% CI [−617.4, −310.84]; standardized *β* = −0.37; Fig. 4*C*). None of the additional predictors, including hearing ability in noise, fearfulness, or performance in the visual emotion recognition tasks, were significantly associated with fiber density in the PUL–BLA pathway.

### Fiber density in the primary auditory route (vMGB–PAC) is associated with better hearing ability but not fearfulness

**Figure 5.**
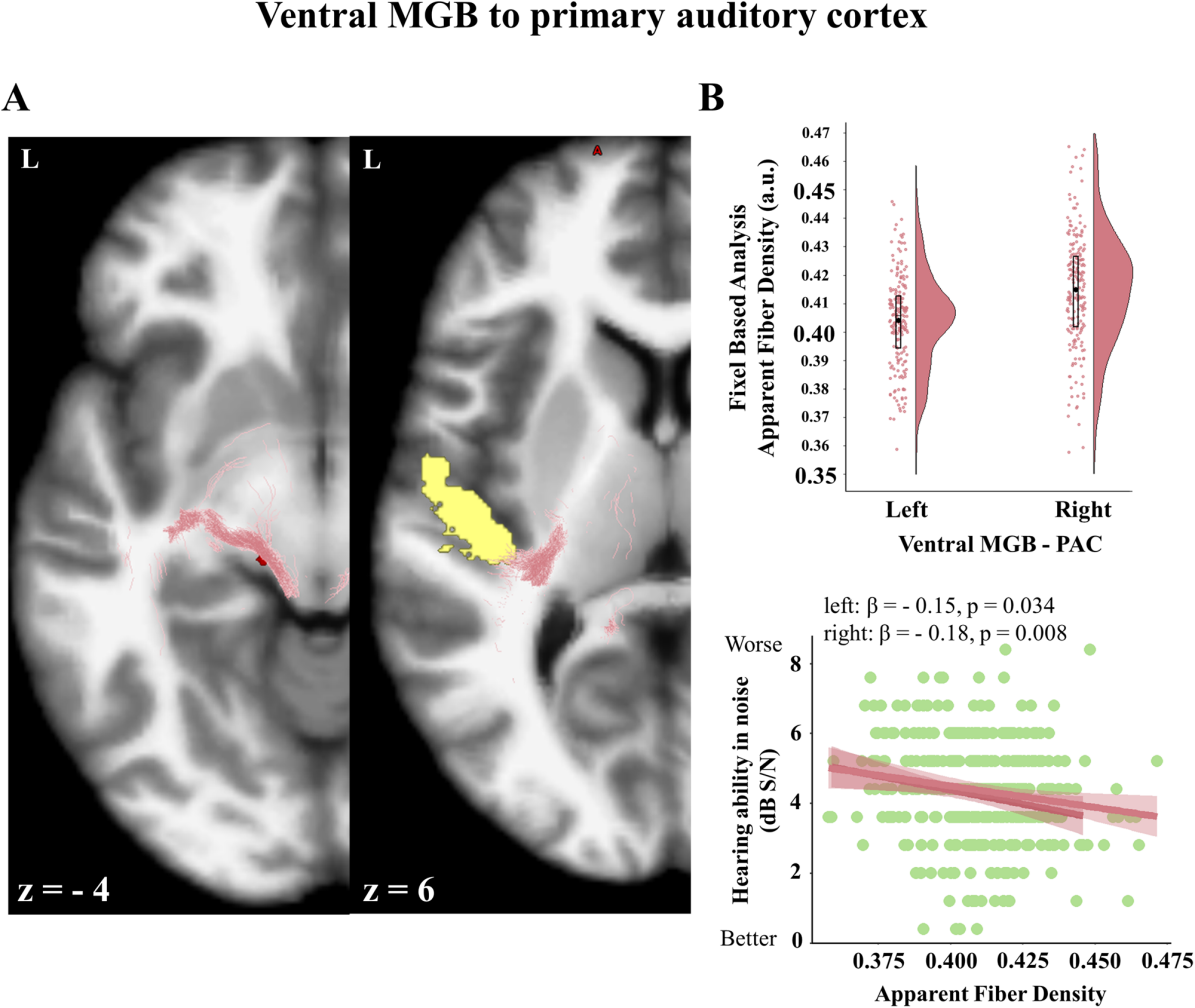
Probabilistic tractography reconstruction of the thalamocortical auditory pathway between the vMGB and the PAC. ***A***, Axial slices at stereotaxic levels *z* = −4, 6 in a representative subject. Fibers reconstructed between the vMGB and the PAC (vMGB in red, PAC in yellow, streamlines in pink). ***B***, Top, Distribution of the apparent fiber density from FBA showing rightward hemispheric asymmetry (*p* < 0.001). Mean values are indicated on the boxplots. ***B***, Bottom, Apparent fiber density for the vMGB–PAC tract correlated negatively with participants’ hearing ability in noise thresholds bilaterally, expressed in dB S/N. Possible scores range from −2.0 to 26.0 dB S/N, with lower scores indicating better performance.

Finally, as a control analysis, we examined the thalamocortical auditory pathway, which constitutes the primary route for auditory processing but does not include direct amygdala projections. For that, we reconstructed streamlines connecting the vMGB to the PAC and found that all participants exhibited at least one connecting streamline bilaterally (number of streamlines; left, *M* = 11,030.74; SD = 782.54; right, *M* = 1,557.79; SD = 2,194.02). In turn, macro- and microstructural fiber density metrics exhibited reverse lateralization profiles. While streamline fiber density was strongly left-sided (left, *M* = 3,934.98; SD = 598.43; right, *M* = 686.35; SD = 648.73; *b* = 3,240.95; SE = 62.41; *t*_(193)_ = −51.93; *p* < 0.001; 95% CI [−3,363.28, −3,118.63]; standardized *β* = −1.87), consistent with measurements in previous tracts, apparent fiber density was higher in the right (*M* = 0.41; SD = 0.02) versus left (*M* = 0.40; SD = 0.02) hemisphere (*b* = 0.011; SE = 0.0009; *t_(_*_193)_ = 11.64; *p* < 0.001; 95% CI [0.47, 0.66]; standardized *β* = 0.56). Please note that, although the absolute numerical difference between hemispheres appears small, apparent fiber density values vary within a narrow range, and, therefore, even subtle mean differences can represent consistent effects across participants. Lastly, our linear mixed model revealed an association of hearing ability in noise with the apparent fiber density of the vMGB–PAC pathway (*b* = −0.0028; SE = 0.0001; *t*_(239)_ = −2.14; *p* = 0.034; 95% CI [−0.0054, −0.0002]; standardized *β* = −0.15), consistent across both hemispheres (left, *β* = −0.15; 95% CI [−0.0054, −0.0002]; *p* = 0.034; right, *β* = −0.18; 95% CI [−0.0061, −0.00092]; *p* = 0.008; Fig. 5). Thus, higher fiber density in this pathway was associated with better hearing ability, supporting its role in auditory function. As expected, no significant interactions were observed between this pathway and fear-related scores.

## Discussion

Here we provide converging human evidence for a direct subcortical pathway connecting the IC to the amygdala via the MGB of the thalamus, showing unprecedented detail and alignment with behavioral measures of fearful feelings and auditory function. This pathway may be analog to the subcortical auditory “low road” for fear described in nonhuman species and may operate alongside the visual route previously identified in humans.

Using DWI in 200 HCP participants and applying probabilistic tractography and FBA, we successfully reconstructed an IC–MGB–BLA pathway, with white matter fiber density correlating with participants' reported emotions of fear. The robustness of the IC–MGB–BLA tract identified in nearly all participants underscores its structural consistency. An anatomical MGB–amygdala link has previously been reported in humans (Keifer et al., 2015), although without explicit evidence for its functional relevance. Our findings extend this work by showing that macrostructural fiber density of the IC–MGB–BLA pathway is associated with fear (LeDoux et al., 1984; Doron and LeDoux, 1999). While the fearfulness score used in our study reflects a transient state (rather than a stable trait), prior work has also reported associations between transient emotional states and structural connectivity (McFadyen et al., 2019; Tang et al., 2020). Similarly, affective states (i.e., state anxiety) are robustly associated with amygdala responsivity to threat (Bishop et al., 2004; Ewbank et al., 2009, 2010), supporting that transient emotional states can be linked to both amygdala function and its structural connectivity. Furthermore, although this was not a primary focus of our study, complementary analyses using trait-based indices showed no significant correlations with fiber density in any of the pathways examined (Supplemental Material, Text S2)*.* Moreover, microstructural density along the reconstructed tract showed an association with hearing ability in noise, such that higher tract integrity related to better auditory perception under degraded acoustic conditions. While speech-in-noise tasks may also involve higher-order auditory mechanisms, their performance depends critically on the integrity of early auditory processing (Dias et al., 2021). Thus, this association supports a contribution of the pathway to auditory function, besides its involvement in emotion. Importantly, the IC–MGB–BLA pathway has been shown to be a key component of the aversion system in rodents (LeDoux et al., 1984, 1986; Bordi and LeDoux, 1994). The BLA is a key structure for fear learning (Fanselow and LeDoux, 1999; Blair et al., 2003) and for the detection of affectively significant stimuli (Wenstrup et al., 2020). In turn, IC and MGB are not only primary auditory relays (Doron and LeDoux, 1999; Bartlett, 2013) but may also have a more direct role in aversive sound detection (Brandão et al., 1988, 2001) and auditory fear conditioning (LeDoux et al., 1990; Lanuza et al., 2004).

The observation that fiber density in both IC–MGB–BLA and MGB–BLA tracts consistently predicted fearfulness and hearing ability in noise provides convergent evidence for the reliability of these associations (Supplemental Material, Text S1). This consistency indicates that the observed relationships likely reflect a stable characteristic of the subcortical pathway, rather than tract-specific or methodological variability. The affective specificity of the IC–MGB–BLA pathway was further supported by an additional analysis incorporating a positive affect measure, which showed no association with fiber density in this pathway (Supplemental Material, Text S3).

Our control analysis of the vMGB to PAC tract, corresponding to the thalamocortical auditory pathway, revealed, as expected, a positive association with hearing ability in noise, but no correlation with fearfulness. This functional dissociation underscores the potential specialization of subcortical auditory inputs to amygdala in emotional function, distinct from the perceptual role of the lemniscal route.

In contrast, another, more direct, tract involving IC–BLA connection was tested, as it was previously reported in humans (Crippa et al., 2010). However, such a connection failed to reach statistical reliability and could only be reconstructed in a small subset of participants. The tract described in Crippa et al. (2010) may thus reflect streamlines passing through the MGB, potentially conflating overlapping trajectories. Other connections between IC and amygdala have been described in bats (Marsh et al., 2002), although these correspond to amygdala outputs to IC. While tractography does not allow direct inference of directionality, our bidirectional modeling captured potential streamlines in both directions. The absence of reliable findings makes the existence of such a connection unlikely.

Both the structure and functional relevance of subcortical pathways may vary across individuals, potentially reflecting primate-specific evolutionary reorganization (McFadyen et al., 2020). Other direct auditory inputs to the amygdala may also exist but remain untested in humans. For example, animal studies describe a dorsal cochlear nucleus–MGB projection that bypasses the IC (Malmierca et al., 2002), but this pathway could not be assessed here due to the small size of the cochlear nucleus and the limitations of diffusion imaging for such regions (Zhang et al., 2019).

Additionally, we explored a PUL–BLA tract encompassing its anterior and medial subdivisions. While a pulvinar–amygdala pathway has been primarily associated with visual and emotion processing (Tamietto et al., 2012; McFadyen et al., 2019), anatomical and electrophysiological evidence suggests that its anteromedial subregions may also receive auditory input, motivating their inclusion (Froesel et al., 2021; Vittek et al., 2023). This pathway emerged as the most robust among those reconstructed, with higher fiber density in this tract being associated with slower emotion recognition in fearful faces. This finding aligns with evidence linking stronger amygdala responses to slower behavioral reactions during visual emotion processing (Domínguez-Borràs et al., 2019) and supports the role of this pathway in evaluating fearful facial expressions, consistent with prior work in the inferior pulvinar (McFadyen et al., 2019). This suggests that this pathway may be associated with the visual subcortical route. However, a complementary analysis using the inferior pulvinar showed no correlations with fearfulness (Supplemental Material, Text S4). Notably, we found no explicit evidence for this tract to be associated with auditory function. Future research incorporating more targeted behavioral measures of auditory emotion may clarify its modality-specific role.

Interestingly, both auditory and affective behavioral measures related to different fiber density metrics: hearing ability in noise correlated with fixel-based fiber density, whereas fearfulness correlated with streamline fiber density. This likely reflects the fact that hearing ability in noise may rely on fine-grained auditory encoding and thus may relate more strongly to local microstructural axonal properties. In contrast, fearfulness may depend more strongly on the integrity of the full thalamo-amygdala pathway. These findings indicate that micro- and macrostructural measures may capture complementary aspects of pathway function. On the other hand, all reconstructed pathways showed hemispheric asymmetry, with stronger fiber density and structure–behavior associations in the left hemisphere. This pattern may be consistent with current models of auditory processing emphasizing a left-hemispheric sensitivity to acoustic features particularly relevant for detecting biologically salient sounds, such as rapid temporal modulations (Galaburda et al., 1994; Flinker et al., 2019; Albouy et al., 2020). This acoustic feature is preferentially encoded in the magnocellular division of the MGB (Winer and Morest, 1983; Trussell, 1997; Winer et al., 1999), specifically in the left hemisphere in humans (Galaburda et al., 1994), and magnocellular MGB corresponds to the origin of the MGB–BLA pathway in nonhuman animals (LeDoux et al., 1990). In addition, human lesion and functional studies indicate a preferential role of the left amygdala in processing vocal emotions and speech-related threat cues (Anderson and Phelps, 2001; Frühholz et al., 2015; Pannese et al., 2016). Together, these converging findings support the leftward asymmetry observed in our study, while its precise link to auditory emotional processing needs further investigation.

Finally, diffusion tractography has inherent limitations, including false positives and negatives, which constrain its ability to confirm specific pathways (Jbabdi and Johansen-Berg, 2011). Histological methods will be essential for validating these connections in the human brain. In addition, our ROIs were derived from heterogeneous sources (cytoarchitectonic, probabilistic, and functional), given our need to use anatomically detailed atlases suited for each structure. Although this may introduce variability in the results, we minimized its impact by applying identical preprocessing, registration, and tractography procedures across all ROIs, and the convergence of results across pathways suggests that our findings were robust to these differences. A further limitation concerns the affective measure used. The fearfulness index reflects a transient state rather than a stable trait. Although state measures are closely linked to amygdala responsivity (Bishop et al., 2004; Ewbank et al., 2009, 2010), future work should systematically characterize the associations of both state- and trait-based constructs with thalamo-amygdala connectivity. Finally, hearing ability in noise may remain an indirect proxy of low-level or subcortical auditory-affective processing. More targeted behavioral tasks will be necessary to characterize the functional role of the IC–MGB–BLA pathway.

In sum, we provide anatomical evidence for a direct subcortical auditory pathway to the amygdala in humans, associated with individual differences in fearfulness and auditory processing. These findings extend current models of emotion processing by suggesting the presence of multiple functional “low roads” for fear in humans, with potentially distinct sensory specializations but converging affective roles. Our study may offer new perspectives on the integration of the auditory and visual systems in affective processes and may provide a framework for future clinical research on affective dysregulation.

## References

[B1] Aggleton JP, Burton MJ, Passingham RE (1980) Cortical and subcortical afferents to the amygdala of the rhesus monkey (*Macaca mulatta*). Brain Res 190:347–368. 10.1016/0006-8993(80)90279-6 6768425

[B2] Albouy P, Benjamin1 L, Morillon B, Zatorre RJ (2020) Distinct sensitivity to spectrotemporal modulation supports brain asymmetry for speech and melody. Science 367:1043–1047. 10.1126/SCIENCE.AAZ3468 32108113

[B3] Amunts K, Kedo O, Kindler M, Pieperhoff P, Mohlberg H, Shah NJ, Habel U, Schneider F, Zilles K (2005) Cytoarchitectonic mapping of the human amygdala, hippocampal region and entorhinal cortex: intersubject variability and probability maps. Anat Embryol 210:343–352. 10.1007/S00429-005-0025-5 16208455

[B4] Anderson AK, Phelps EA (2001) Lesions of the human amygdala impair enhanced perception of emotionally salient events. Nature 411:305–309. 10.1038/35077083 11357132

[B6] Andersson JLR, Sotiropoulos SN (2016) An integrated approach to correction for off-resonance effects and subject movement in diffusion MR imaging. Neuroimage 125:1063–1078. 10.1016/J.NEUROIMAGE.2015.10.019 26481672 PMC4692656

[B5] Andersson JLR, Skare S, Ashburner J (2003) How to correct susceptibility distortions in spin-echo echo-planar images: application to diffusion tensor imaging. Neuroimage 20:870–888. 10.1016/S1053-8119(03)00336-7 14568458

[B7] Armony JL, Servan-Schreiber D, Cohen JD, LeDoux JE (1997) Computational modeling of emotion: explorations through the anatomy and physiology of fear conditioning. Trends Cogn Sci 1:28–34. 10.1016/S1364-6613(97)01007-3 21223850

[B8] Barron DS, Eickhoff SB, Clos M, Fox PT (2015) Human pulvinar functional organization and connectivity. Hum Brain Mapp 36:2417–2431. 10.1002/HBM.22781 25821061 PMC4782796

[B9] Bartlett EL (2013) The organization and physiology of the auditory thalamus and its role in processing acoustic features important for speech perception. Brain Lang 126:29–48. 10.1016/J.BANDL.2013.03.003 23725661 PMC3707394

[B10] Bates D, Mächler M, Bolker B, Walker S (2015) Fitting linear mixed-effects models using lme4. J Stat Softw 67:1–48. 10.18637/jss.v067.i01

[B11] Bernstein JGW, Danielsson H, Hällgren M, Stenfelt S, Rönnberg J, Lunner T (2016) Spectrotemporal modulation sensitivity as a predictor of speech-reception performance in noise with hearing aids. Trends Hear 20:2331216516670387. 10.1177/2331216516670387 27815546 PMC5098798

[B12] Bianciardi M, Cauzzo S, Toschi N, Koley S, García-Gomar M, Singh K (2023) The Brainstem Navigator: a toolkit to investigate brainstem nuclei structure, function, and connectivity in living humans. In 2023 ISMRM & ISMRT Annual Meeting.

[B13] Bishop SJ, Duncan J, Lawrence AD (2004) State anxiety modulation of the amygdala response to unattended threat-related stimuli. J Neurosci 24:10364–10368. 10.1523/JNEUROSCI.2550-04.2004 15548650 PMC6730310

[B14] Blair HT, Tinkelman A, Moita MAP, LeDoux JE (2003) Associative plasticity in neurons of the lateral amygdala during auditory fear conditioning. Ann N Y Acad Sci 985:485–487. 10.1111/J.1749-6632.2003.TB07106.X 12724183

[B15] Bordi F, LeDoux JE (1994) Response properties of single units in areas of rat auditory thalamus that project to the amygdala - I. Acoustic discharge patterns and frequency receptive fields. Exp Brain Res 98:261–274. 10.1007/BF00228414, 8050512

[B16] Bourgeois A, Guedj C, Carrera E, Vuilleumier P (2020) Pulvino-cortical interaction: an integrative role in the control of attention. Neurosci Biobehav Rev 111:104–113. 10.1016/J.NEUBIOREV.2020.01.005 31972202

[B17] Brandão ML, Tomaz C, Leão Borges PC, Coimbra NC, Bagri A (1988) Defense reaction induced by microinjections of bicuculline into the inferior colliculus. Physiol Behav 44:361–365. 10.1016/0031-9384(88)90038-8 2851846

[B18] Brandão ML, Coimbra NC, Osaki MY (2001) Changes in the auditory-evoked potentials induced by fear-evoking stimulations. Physiol Behav 72:365–372. 10.1016/S0031-9384(00)00418-2 11274679

[B19] Carr JA (2015) I’ll take the low road: the evolutionary underpinnings of visually triggered fear. Front Neurosci 9:414. 10.3389/fnins.2015.00414 26578871 PMC4624861

[B20] Celeghin A, Bagnis A, Diano M, Méndez CA, Costa T, Tamietto M (2019) Functional neuroanatomy of blindsight revealed by activation likelihood estimation meta-analysis. Neuropsychologia 128:109–118. 10.1016/j.neuropsychologia.2018.06.007 29894718

[B21] Chandrasekaran B, Hornickel J, Skoe E, Nicol T, Kraus N (2009) Context-dependent encoding in the human auditory brainstem relates to hearing speech in noise: implications for developmental dyslexia. Neuron 64:311–319. 10.1016/j.neuron.2009.10.006 19914180 PMC2778610

[B22] Crippa A, Lanting CP, van Dijk P, Roerdink JBTM (2010) A diffusion tensor imaging study on the auditory system and tinnitus. Open Neuroimag J 4:16–25. 10.2174/1874440001004010016 20922048 PMC2948149

[B23] Day-Brown JD, Wei H, Chomsung RD, Petry HM, Bickford ME (2010) Pulvinar projections to the striatum and amygdala in the tree shrew. Front Neuroanat 4:4–143. 10.3389/FNANA.2010.00143/BIBTEX 21120139 PMC2991220

[B24] Dhollander T, et al. (2021) Fixel-based analysis of diffusion MRI: methods, applications, challenges and opportunities. Neuroimage 241:118417. 10.1016/j.neuroimage.2021.118417 34298083

[B25] Dias JW, McClaskey CM, Harris KC (2021) Early auditory cortical processing predicts auditory speech in noise identification and lipreading. Neuropsychologia 161:108012. 10.1016/J.NEUROPSYCHOLOGIA.2021.108012 34474065 PMC8487996

[B27] Domínguez-Borràs J, Vuilleumier P (2022) Amygdala function in emotion, cognition, and behavior. Handb Clin Neurol 187:359–380. 10.1016/B978-0-12-823493-8.00015-8 35964983

[B26] Domínguez-Borràs J, et al. (2019) Human amygdala response to unisensory and multisensory emotion input: no evidence for superadditivity from intracranial recordings. Neuropsychologia 131:9–24. 10.1016/j.neuropsychologia.2019.05.027 31158367

[B28] Doron NN, LeDoux JE (1999) Organization of projections to the lateral amygdala from auditory and visual areas of the thalamus in the rat. J Comp Neurol 412:383–409. 10.1002/(SICI)1096-9861(19990927)412:3<383::AID-CNE2>3.0.CO;2-5 10441229

[B29] Eickhoff SB, Stephan KE, Mohlberg H, Grefkes C, Fink GR, Amunts K, Zilles K (2005) A new SPM toolbox for combining probabilistic cytoarchitectonic maps and functional imaging data. Neuroimage 25:1325–1335. 10.1016/J.NEUROIMAGE.2004.12.034 15850749

[B30] Elorette C, Forcelli PA, Saunders RC, Malkova L (2018) Colocalization of tectal inputs with amygdala-projecting neurons in the macaque pulvinar. Front Neural Circuits 12:91. 10.3389/FNCIR.2018.00091 30405362 PMC6207581

[B31] Ewbank MP, Lawrence AD, Passamonti L, Keane J, Peers PV, Calder AJ (2009) Anxiety predicts a differential neural response to attended and unattended facial signals of anger and fear. Neuroimage 44:1144–1151. 10.1016/J.NEUROIMAGE.2008.09.056 18996489

[B32] Ewbank MP, Fox E, Calder AJ (2010) The interaction between gaze and facial expression in the amygdala and extended amygdala is modulated by anxiety. Front Hum Neurosci 4:1272. 10.3389/FNHUM.2010.00056/XML PMC2906373 20661452

[B33] Fanselow MS, LeDoux JE (1999) Why we think plasticity underlying Pavlovian fear conditioning occurs in the basolateral amygdala. Neuron 23:229–232. 10.1016/S0896-6273(00)80775-8 10399930

[B34] Flinker A, Doyle WK, Mehta AD, Devinsky O, Poeppel D (2019) Spectrotemporal modulation provides a unifying framework for auditory cortical asymmetries. Nat Hum Behav 3:393–405. 10.1038/s41562-019-0548-z 30971792 PMC6650286

[B35] Froesel M, Cappe C, Ben Hamed S (2021) NC-ND license a multisensory perspective onto primate pulvinar functions. Neurosci Biobehav Rev 125:231–243. 10.1016/j.neubiorev.2021.02.043 33662442

[B36] Froesel M, Gacoin M, Clavagnier S, Hauser M, Goudard Q, Ben Hamed S (2024) Macaque claustrum, pulvinar and putative dorsolateral amygdala support the cross-modal association of social audio-visual stimuli based on meaning. Eur J Neurosci 59:3203–3223. 10.1111/EJN.16328 38637993

[B37] Frühholz S, Hofstetter C, Cristinzio C, Saj A, Seeck M, Vuilleumier P, Grandjean D (2015) Asymmetrical effects of unilateral right or left amygdala damage on auditory cortical processing of vocal emotions. Proc Natl Acad Sci U S A 112:1583–1588. 10.1073/PNAS.1411315112 25605886 PMC4321266

[B38] Galaburda AM, Menard MT, Rosen GD (1994) Evidence for aberrant auditory anatomy in developmental dyslexia. Proc Natl Acad Sci U S A 91:8010. 10.1073/PNAS.91.17.8010 8058748 PMC44534

[B39] Glasser MF, et al. (2013) The minimal preprocessing pipelines for the human connectome project. Neuroimage 80:105–124. 10.1016/j.neuroimage.2013.04.127 23668970 PMC3720813

[B40] Greve DN, Fischl B (2009) Accurate and robust brain image alignment using boundary-based registration. Neuroimage 48:63–72. 10.1016/J.NEUROIMAGE.2009.06.060 19573611 PMC2733527

[B41] Grieve KL, Acuña C, Cudeiro J (2000) The primate pulvinar nuclei: vision and action. Trends Neurosci 23:35–39. 10.1016/S0166-2236(99)01482-4 10631787

[B42] Homman-Ludiye J, Bourne JA (2019) The medial pulvinar: function, origin and association with neurodevelopmental disorders. J Anat 235:507–520. 10.1111/joa.12932 30657169 PMC6704239

[B43] Jbabdi S, Johansen-Berg H (2011) Tractography: where do we go from here? Brain Connect 1:169–183. 10.1089/brain.2011.0033 22433046 PMC3677805

[B44] Jenkinson M, Bannister P, Brady M, Smith S (2002) Improved optimization for the robust and accurate linear registration and motion correction of brain images. Neuroimage 17:825–841. 10.1016/S1053-8119(02)91132-8 12377157

[B45] Jenkinson M, Beckmann CF, Behrens TEJ, Woolrich MW, Smith SM (2012) FSL. Neuroimage 62:782–790. 10.1016/J.NEUROIMAGE.2011.09.015 21979382

[B46] Jeurissen B, Tournier JD, Dhollander T, Connelly A, Sijbers J (2014) Multi-tissue constrained spherical deconvolution for improved analysis of multi-shell diffusion MRI data. Neuroimage 103:411–426. 10.1016/J.NEUROIMAGE.2014.07.061 25109526

[B47] Keifer OP, Gutman DA, Hecht EE, Keilholz SD, Ressler KJ (2015) A comparative analysis of mouse and human medial geniculate nucleus connectivity: a DTI and anterograde tracing study. Neuroimage 105:53–66. 10.1016/j.neuroimage.2014.10.047 25450110 PMC4262636

[B48] Khalil V, Faress I, Mermet-Joret N, Kerwin P, Yonehara K, Nabavi S (2023) Subcortico-amygdala pathway processes innate and learned threats. Elife 12:e85459. 10.7554/ELIFE.85459 37526552 PMC10449383

[B49] Kiwitz K, Brandstetter A, Schiffer C, Bludau S, Mohlberg H, Omidyeganeh M, Massicotte P, Amunts K (2022) Cytoarchitectonic maps of the human metathalamus in 3D space. Front Neuroanat 16:837485. 10.3389/FNANA.2022.837485 35350721 PMC8957853

[B50] Kragel PA, Čeko M, Theriault J, Chen D, Satpute AB, Wald LW, Lindquist MA, Feldman Barrett L, Wager TD (2021) A human colliculus-pulvinar-amygdala pathway encodes negative emotion. Neuron 109:2404–2412.e5. 10.1016/j.neuron.2021.06.001 34166604 PMC8349850

[B51] Lanuza E, Nader K, Ledoux JE (2004) Unconditioned stimulus pathways to the amygdala: effects of posterior thalamic and cortical lesions on fear conditioning. Neuroscience 125:305–315. 10.1016/j.neuroscience.2003.12.034 15062974

[B52] LeDoux JE, Sakaguchi A, Reis DJ (1984) Subcortical efferent projections of the medial geniculate nucleus mediate emotional responses conditioned to acoustic stimuli. J Neurosci 4:683–698. 10.1523/JNEUROSCI.04-03-00683.1984 6707732 PMC6564820

[B53] LeDoux JE, Iwata J, Pearl D, Reis DJ (1986) Disruption of auditory but not visual learning by destruction of intrinsic neurons in the rat medial geniculate body. Brain Res 371:395–399. 10.1016/0006-8993(86)90383-5 3697769

[B54] LeDoux JE, Farb C, Ruggiero DA (1990) Topographic organization of neurons in the acoustic thalamus that project to the amygdala. J Neurosci 10:1043–1054. 10.1523/JNEUROSCI.10-04-01043.1990 2158523 PMC6570207

[B55] LeDoux JE (1996) The emotional brain: the mysterious underpinnings of emotional life. New York: Simon & Schuster.

[B56] Lee CC (2012) Thalamic and cortical pathways supporting auditory processing. Brain Lang 126:22–28. 10.1016/J.BANDL.2012.05.004 22728130 PMC3483386

[B57] Lenth R (2025) *emmeans: Estimated Marginal Means, aka Least-Squares Means. R package version 1.11.1-00001*. https://github.com/rvlenth/emmeans

[B58] Malmierca MS, Merchán MA, Henkel CK, Oliver DL (2002) Direct projections from cochlear nuclear complex to auditory thalamus in the rat. J Neurosci 22:10891–10897. 10.1523/JNEUROSCI.22-24-10891.2002 12486183 PMC6758437

[B59] Marsh RA, Fuzessery ZM, Grose CD, Wenstrup JJ (2002) Projection to the inferior colliculus from the basal nucleus of the amygdala. J Neurosci 22:10449–10460. 10.1523/JNEUROSCI.22-23-10449.2002 12451144 PMC6758740

[B60] McFadyen J, Mattingley JB, Garrido MI (2019) An afferent white matter pathway from the pulvinar to the amygdala facilitates fear recognition. Elife 8:e40766. 10.7554/eLife.40766 30648533 PMC6335057

[B61] McFadyen J, Dolan RJ, Garrido MI (2020) The influence of subcortical shortcuts on disordered sensory and cognitive processing. Nat Rev Neurosci 21:264–276. 10.1038/s41583-020-0287-1 32269315

[B62] Mihai PG, Moerel M, De Martino F, Trampel R, Kiebel S, Von Kriegstein K (2019) Modulation of tonotopic ventral medial geniculate body is behaviorally relevant for speech recognition. Elife 8:e44837. 10.7554/eLife.44837.001 31453811 PMC6711666

[B63] Morris DM, Embleton KV, Parker GJM (2008) Probabilistic fibre tracking: differentiation of connections from chance events. Neuroimage 42:1329–1339. 10.1016/J.NEUROIMAGE.2008.06.012 18619548

[B64] Nakataki M, et al. (2016) Glucocorticoid administration improves aberrant fear-processing networks in spider phobia. Neuropsychopharmacology 42:485–494. 10.1038/npp.2016.207 27644128 PMC5399241

[B65] Pannese A, Grandjean D, Frühholz S (2016) Amygdala and auditory cortex exhibit distinct sensitivity to relevant acoustic features of auditory emotions. Cortex 85:116–125. 10.1016/J.CORTEX.2016.10.013 27855282

[B66] Parbery-Clark A, Marmel F, Bair J, Kraus N (2011) What subcortical–cortical relationships tell US about processing speech in noise. Eur J Neurosci 33:549–557. 10.1111/J.1460-9568.2010.07546.X 21255123

[B67] Pilkonis PA, Choi SW, Reise SP, Stover AM, Riley WT, Cella D (2011) Item banks for measuring emotional distress from the patient-reported outcomes measurement information system (PROMIS®): depression, anxiety, and anger. Assessment 18:263–283. 10.1177/1073191111411667 21697139 PMC3153635

[B68] Pilkonis PA, et al. (2013) Assessment of self-reported negative affect in the NIH toolbox. Psychiatry Res 206:88–97. 10.1016/j.psychres.2012.09.034 23083918 PMC3561498

[B69] Rafal RD, Koller K, Bultitude JH, Mullins P, Ward R, Mitchell AS, Bell AH (2015) Connectivity between the superior colliculus and the amygdala in humans and macaque monkeys: virtual dissection with probabilistic DTI tractography. J Neurophysiol 114:1947–1962. 10.1152/JN.01016.2014/SUPPL_FILE/VIDEO_S2.GIF 26224780 PMC4579293

[B70] Raffelt DA, Smith RE, Ridgway GR, Tournier JD, Vaughan DN, Rose S, Henderson R, Connelly A (2015) Connectivity-based fixel enhancement: whole-brain statistical analysis of diffusion MRI measures in the presence of crossing fibres. Neuroimage 117:40–55. 10.1016/J.NEUROIMAGE.2015.05.039 26004503 PMC4528070

[B71] Raffelt DA, Tournier JD, Smith RE, Vaughan DN, Jackson G, Ridgway GR, Connelly A (2017) Investigating white matter fibre density and morphology using fixel-based analysis. Neuroimage 144:58–73. 10.1016/J.NEUROIMAGE.2016.09.029 27639350 PMC5182031

[B72] Raffelt D, Tournier JD, Rose S, Ridgway GR, Henderson R, Crozier S, Salvado O, Connelly A (2012) Apparent fibre density: a novel measure for the analysis of diffusion-weighted magnetic resonance images. Neuroimage 59:3976–3994. 10.1016/J.NEUROIMAGE.2011.10.045 22036682

[B73] Salsman JM, et al. (2013) Emotion assessment using the NIH toolbox. Neurology 80:S76. 10.1212/WNL.0B013E3182872E11 23479549 PMC3662334

[B74] Shinonaga Y, Takada M, Mizuno N (1994) Direct projections from the non-laminated divisions of the medial geniculate nucleus to the temporal polar cortex and amygdala in the cat. J Comp Neurol 340:405–426. 10.1002/CNE.903400310 8188859

[B75] Smith RE, Tournier JD, Calamante F, Connelly A (2012) Anatomically-constrained tractography: improved diffusion MRI streamlines tractography through effective use of anatomical information. Neuroimage 62:1924–1938. 10.1016/J.NEUROIMAGE.2012.06.005 22705374

[B76] Smith RE, Tournier JD, Calamante F, Connelly A (2015) SIFT2: enabling dense quantitative assessment of brain white matter connectivity using streamlines tractography. Neuroimage 119:338–351. 10.1016/J.NEUROIMAGE.2015.06.092 26163802

[B77] Tamietto M, Pullens P, De Gelder B, Weiskrantz L, Goebel R (2012) Subcortical connections to human amygdala and changes following destruction of the visual cortex. Curr Biol 22:1449–1455. 10.1016/j.cub.2012.06.006 22748315

[B78] Tamietto M, De Gelder B (2010) Neural bases of the non-conscious perception of emotional signals. Nat Rev Neurosci 11:697–709. 10.1038/NRN2889 20811475

[B79] Tang TY, Luan Y, Jiao Y, Zhang J, Ju SH, Teng GJ (2020) Disrupted amygdala connectivity is associated with elevated anxiety in sensorineural hearing loss. Front Neurosci 14:616348. 10.3389/FNINS.2020.616348/BIBTEX 33362462 PMC7758419

[B80] Tournier JD, Calamante F, Connelly A (2012) MRtrix: diffusion tractography in crossing fiber regions. Int J Imaging Syst Technol 22:53–66. 10.1002/IMA.22005

[B81] Tournier JD, Smith R, Raffelt D, Tabbara R, Dhollander T, Pietsch M, Christiaens D, Jeurissen B, Yeh CH, Connelly A (2019) MRtrix3: a fast, flexible and open software framework for medical image processing and visualisation. Neuroimage 202:116137. 10.1016/j.neuroimage.2019.116137 31473352

[B82] Trussell LO (1997) Cellular mechanisms for preservation of timing in central auditory pathways. Curr Opin Neurobiol 7:487–492. 10.1016/S0959-4388(97)80027-X 9287194

[B83] Van Essen DC, Smith SM, Barch DM, Behrens TE, Yacoub E, Ugurbil K, WU-Minn HCP Consortium (2013) The WU-Minn Human Connectome Project: an overview. Neuroimage 180:62–79. 10.1016/j.neuroimage.2013.05.041 PMC3724347 23684880

[B84] Vittek AL, Juan C, Nowak LG, Girard P, Cappe C (2023) Multisensory integration in neurons of the medial pulvinar of macaque monkey. Cereb Cortex 33:4202–4215. 10.1093/CERCOR/BHAC337 36068947 PMC10110443

[B85] von Kriegstein K, Patterson RD, Griffiths TD (2008) Task-Dependent modulation of medial geniculate body is behaviorally relevant for speech recognition. Curr Biol 18:1855–1859. 10.1016/j.cub.2008.10.052 19062286 PMC2631608

[B86] Vuilleumier P (2005) How brains beware: neural mechanisms of emotional attention. Trends Cogn Sci 9:585–594. 10.1016/J.TICS.2005.10.011 16289871

[B87] Wei P, et al. (2015) Processing of visually evoked innate fear by a non-canonical thalamic pathway. Nat Commun 6:1–13. 10.1038/ncomms7756 PMC4403372 25854147

[B88] Wenstrup JJ, Ghasemahmad Z, Hazlett E, Shanbhag SJ (2020) The amygdala–a Hub of the social auditory brain. In: The senses: a comprehensive reference (Fritzsch B, ed), Vols. 1–7, pp 812–837. London: Elsevier.

[B89] Winer JA, Kelly JB, Larue DT (1999) Neural architecture of the rat medial geniculate body. Hear Res 130:19–41. 10.1016/S0378-5955(98)00216-0 10320097

[B90] Winer JA, Morest DK (1983) The medial division of the medial geniculate body of the cat: implications for thalamic organization. J Neurosci 3:2629–2651. 10.1523/JNEUROSCI.03-12-02629.1983 6655503 PMC6564649

[B91] Zecker SG, et al. (2013) Audition assessment using the NIH toolbox. Neurology 80:45–48. 10.1212/WNL.0B013E3182872DD2 PMC3662344 23479544

[B92] Zhang F, Wu Y, Norton I, Rathi Y, Golby AJ, O’Donnell LJ (2019) Test–retest reproducibility of white matter parcellation using diffusion MRI tractography fiber clustering. Hum Brain Mapp 40:3041–3057. 10.1002/HBM.24579 30875144 PMC6548665

